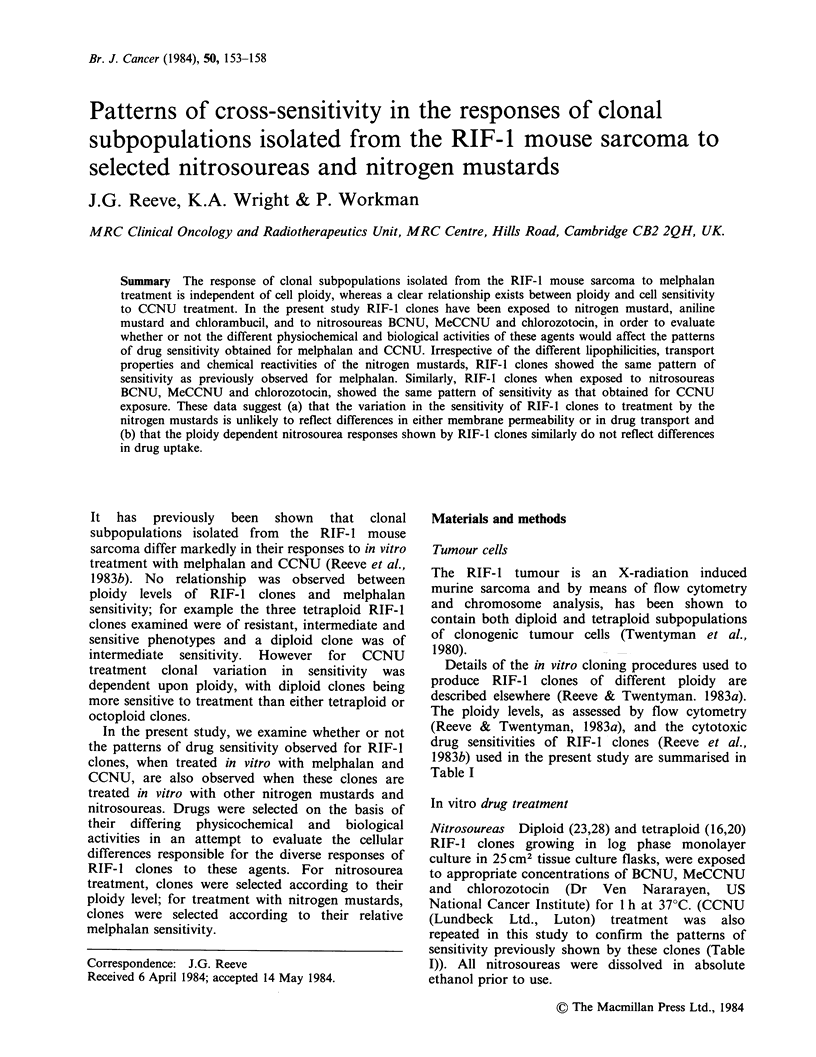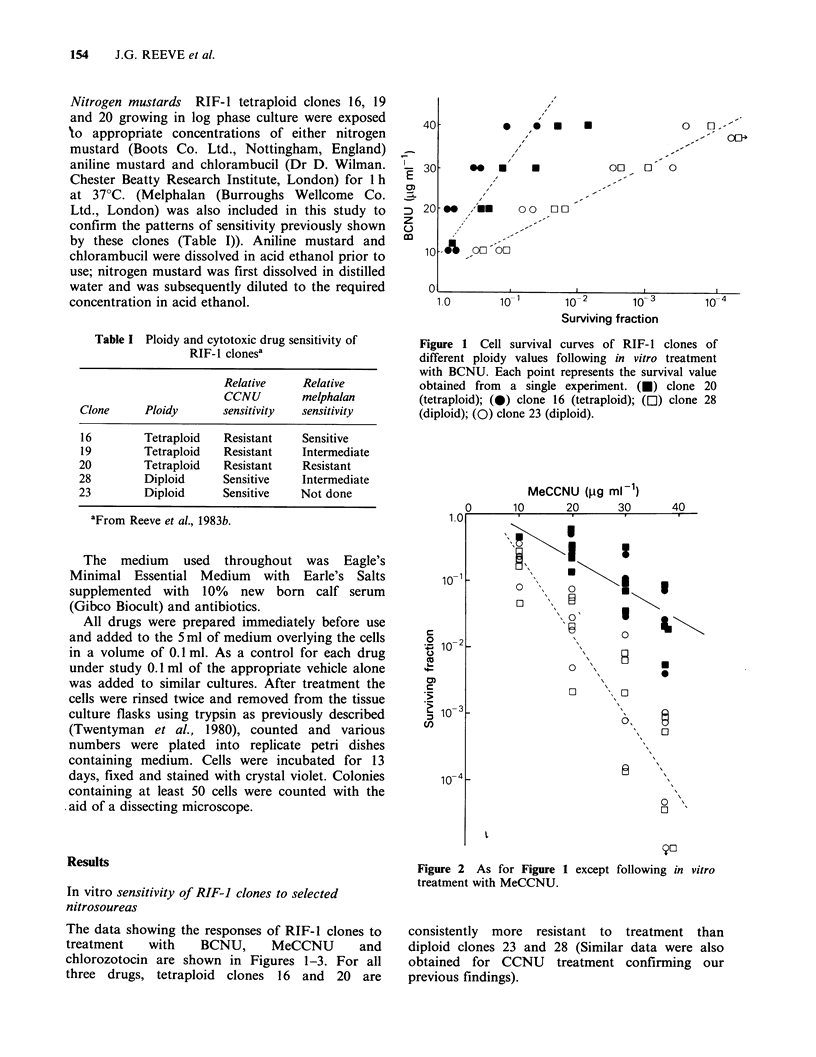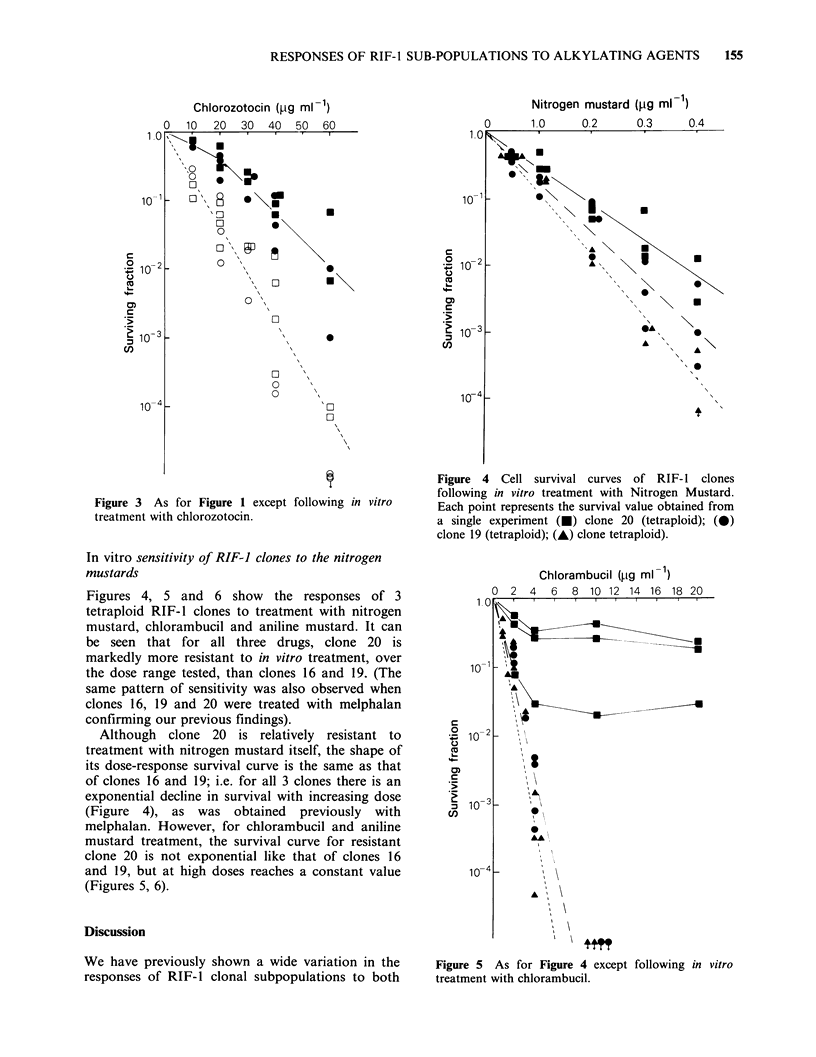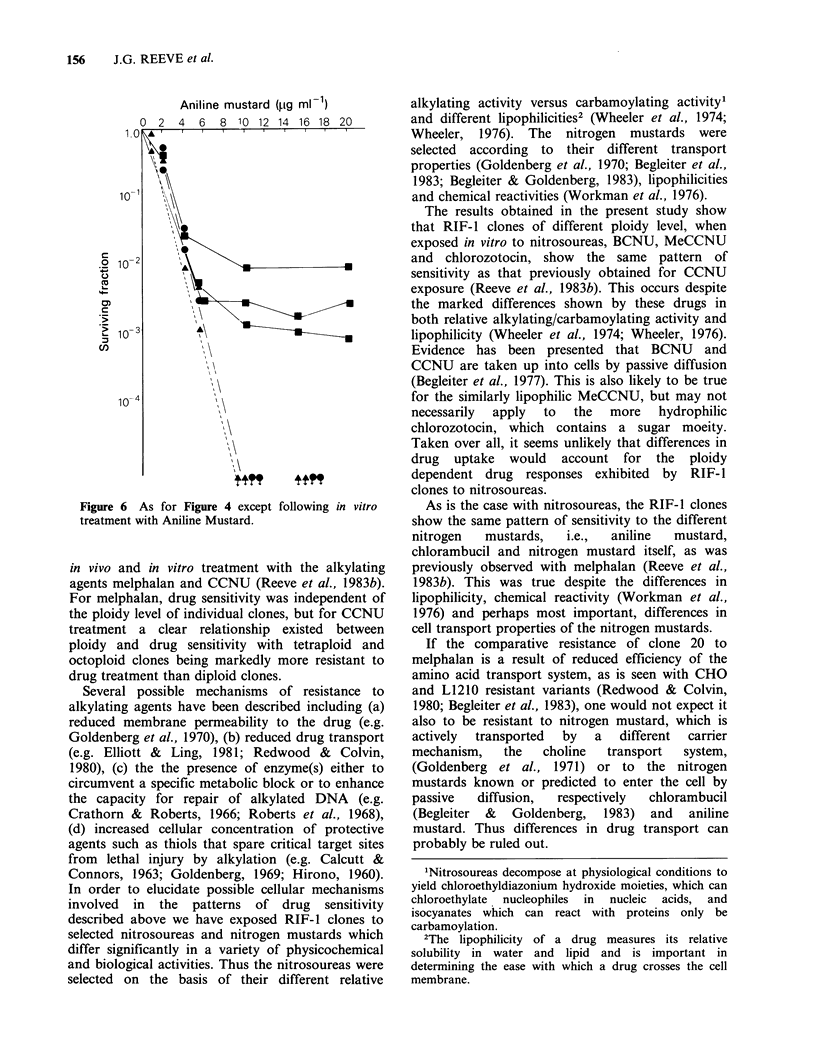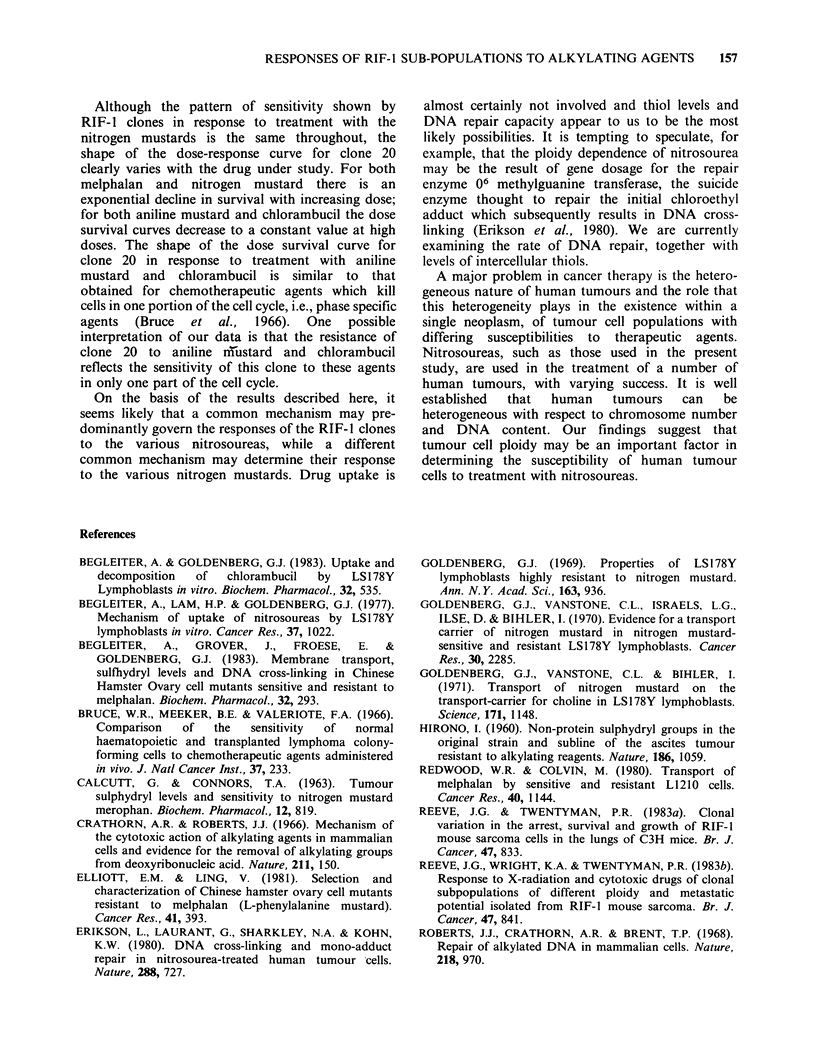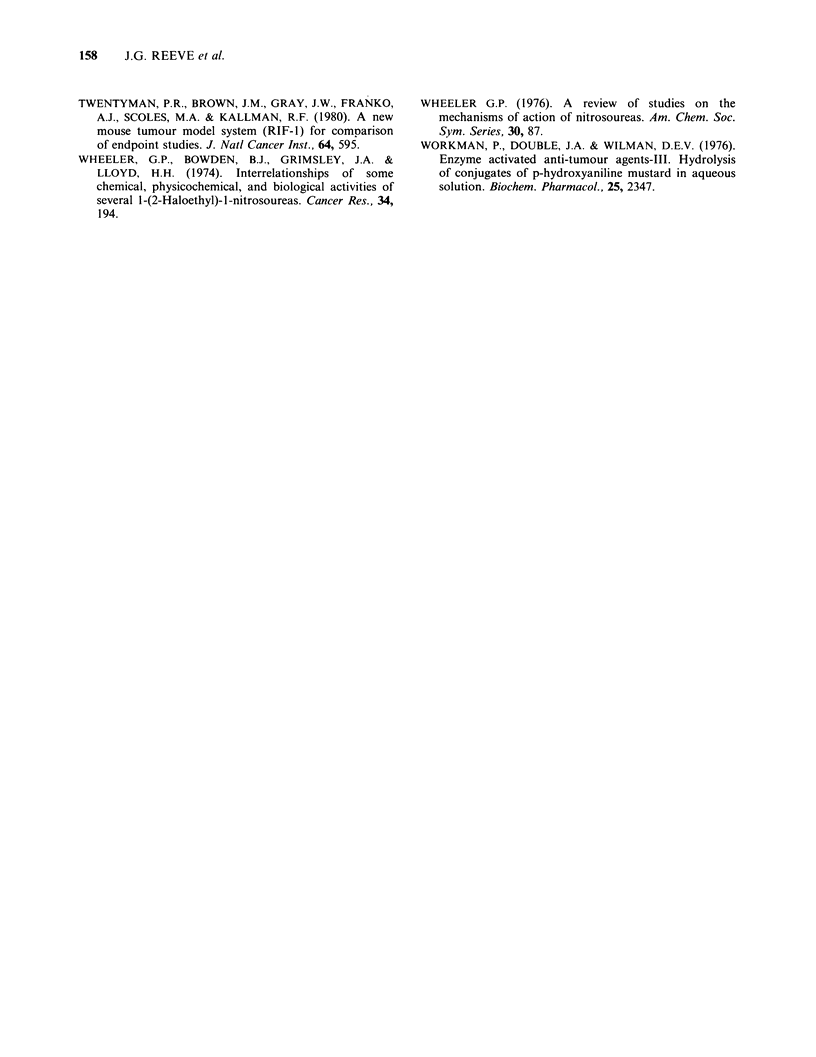# Patterns of cross-sensitivity in the responses of clonal subpopulations isolated from the RIF-1 mouse sarcoma to selected nitrosoureas and nitrogen mustards.

**DOI:** 10.1038/bjc.1984.157

**Published:** 1984-08

**Authors:** J. G. Reeve, K. A. Wright, P. Workman

## Abstract

The response of clonal subpopulations isolated from the RIF-1 mouse sarcoma to melphalan treatment is independent of cell ploidy, whereas a clear relationship exists between ploidy and cell sensitivity to CCNU treatment. In the present study RIF-1 clones have been exposed to nitrogen mustard, aniline mustard and chlorambucil, and to nitrosoureas BCNU, MeCCNU and chlorozotocin, in order to evaluate whether or not the different physiochemical and biological activities of these agents would affect the patterns of drug sensitivity obtained for melphalan and CCNU. Irrespective of the different lipophilicities, transport properties and chemical reactivities of the nitrogen mustards, RIF-1 clones showed the same pattern of sensitivity as previously observed for melphalan. Similarly, RIF-1 clones when exposed to nitrosoureas BCNU, MeCCNU and chlorozotocin, showed the same pattern of sensitivity as that obtained for CCNU exposure. These data suggest (a) that the variation in the sensitivity of RIF-1 clones to treatment by the nitrogen mustards is unlikely to reflect differences in either membrane permeability or in drug transport and (b) that the ploidy dependent nitrosourea responses shown by RIF-1 clones similarly do not reflect differences in drug uptake.


*Br. J. Cancer* (1984), **50**, 153–158

### Patterns of cross-sensitivity in the responses of clonal subpopulations isolated from the RIF-1 mouse sarcoma to selected nitrosoureas and nitrogen mustards

J.G. Reeve, K.A. Wright & P. Workman

*MRC Clinical Oncology and Radiotherapeutics Unit, MRC Centre, Hills Road, Cambridge CB2 2QH, UK.*

**Summary** The response of clonal subpopulations isolated from the RIF-1 mouse sarcoma to melphalan treatment is independent of cell ploidy, whereas a clear relationship exists between ploidy and cell sensitivity to CCNU treatment. In the present study RIF-1 clones have been exposed to nitrogen mustard, aniline mustard and chlorambucil, and to nitrosoureas BCNU, MeCCNU and chlorozotocin, in order to evaluate whether or not the different physiochemical and biological activities of these agents would affect the patterns of drug sensitivity obtained for melphalan and CCNU. Irrespective of the different lipophilicities, transport properties and chemical reactivities of the nitrogen mustards, RIF-1 clones showed the same pattern of sensitivity as previously observed for melphalan. Similarly, RIF-1 clones when exposed to nitrosoureas BCNU, MeCCNU and chlorozotocin, showed the same pattern of sensitivity as that obtained for CCNU exposure. These data suggest (a) that the variation in the sensitivity of RIF-1 clones to treatment by the nitrogen mustards is unlikely to reflect differences in either membrane permeability or in drug transport and (b) that the ploidy dependent nitrosourea responses shown by RIF-1 clones similarly do not reflect differences in drug uptake.

It has previously been shown that clonal subpopulations isolated from the RIF-1 mouse sarcoma differ markedly in their responses to *in vitro* treatment with melphalan and CCNU (Reeve *et al.*, 1983*b*). No relationship was observed between ploidy levels of RIF-1 clones and melphalan sensitivity; for example the three tetraploid RIF-1 clones examined were of resistant, intermediate and sensitive phenotypes and a diploid clone was of intermediate sensitivity. However for CCNU treatment clonal variation in sensitivity was dependent upon ploidy, with diploid clones being more sensitive to treatment than either tetraploid or octoploid clones.

In the present study, we examine whether or not the patterns of drug sensitivity observed for RIF-1 clones, when treated *in vitro* with melphalan and CCNU, are also observed when these clones are treated *in vitro* with other nitrogen mustards and nitrosoureas. Drugs were selected on the basis of their differing physicochemical and biological activities in an attempt to evaluate the cellular differences responsible for the diverse responses of RIF-1 clones to these agents. For nitrosourea treatment, clones were selected according to their ploidy level; for treatment with nitrogen mustards, clones were selected according to their relative melphalan sensitivity.

Correspondence: J.G. Reeve
Received 6 April 1984; accepted 14 May 1984.

### Materials and methods

### Tumour cells

The RIF-1 tumour is an X-radiation induced murine sarcoma and by means of flow cytometry and chromosome analysis, has been shown to contain both diploid and tetraploid subpopulations of clonogenic tumour cells (Twentyman *et al.*, 1980).

Details of the *in vitro* cloning procedures used to produce RIF-1 clones of different ploidy are described elsewhere (Reeve & Twentyman. 1983*a*). The ploidy levels, as assessed by flow cytometry (Reeve & Twentyman, 1983*a*), and the cytotoxic drug sensitivities of RIF-1 clones (Reeve *et al.*, 1983*b*) used in the present study are summarised in Table I

### In vitro *drug treatment*

*Nitrosoureas* Diploid (23,28) and tetraploid (16,20) RIF-1 clones growing in log phase monolayer culture in 25 cm² tissue culture flasks, were exposed to appropriate concentrations of BCNU, MeCCNU and chlorozotocin (Dr Ven Nararayen, US National Cancer Institute) for 1 h at 37°C. (CCNU (Lundbeck Ltd., Luton) treatment was also repeated in this study to confirm the patterns of sensitivity previously shown by these clones (Table I)). All nitrosoureas were dissolved in absolute ethanol prior to use.

© The Macmillan Press Ltd., 1984

*Nitrogen mustards* RIF-1 tetraploid clones 16, 19 and 20 growing in log phase culture were exposed to appropriate concentrations of either nitrogen mustard (Boots Co. Ltd., Nottingham, England) aniline mustard and chlorambucil (Dr D. Wilman. Chester Beatty Research Institute, London) for 1 h at 37°C. (Melphalan (Burroughs Wellcome Co. Ltd., London) was also included in this study to confirm the patterns of sensitivity previously shown by these clones (Table I)). Aniline mustard and chlorambucil were dissolved in acid ethanol prior to use; nitrogen mustard was first dissolved in distilled water and was subsequently diluted to the required concentration in acid ethanol.

**Table I** Ploidy and cytotoxic drug sensitivity of RIF-1 clones[a]

| *Clone* | *Ploidy* | *Relative CCNU sensitivity* | *Relative melphalan sensitivity* |
|---|---|---|---|
| 16 | Tetraploid | Resistant | Sensitive |
| 19 | Tetraploid | Resistant | Intermediate |
| 20 | Tetraploid | Resistant | Resistant |
| 28 | Diploid | Sensitive | Intermediate |
| 23 | Diploid | Sensitive | Not done |

[a]From Reeve *et al.*, 1983*b*.

The medium used throughout was Eagle's Minimal Essential Medium with Earle's Salts supplemented with 10% new born calf serum (Gibco Biocult) and antibiotics.

All drugs were prepared immediately before use and added to the 5 ml of medium overlying the cells in a volume of 0.1 ml. As a control for each drug under study 0.1 ml of the appropriate vehicle alone was added to similar cultures. After treatment the cells were rinsed twice and removed from the tissue culture flasks using trypsin as previously described (Twentyman *et al.*, 1980), counted and various numbers were plated into replicate petri dishes containing medium. Cells were incubated for 13 days, fixed and stained with crystal violet. Colonies containing at least 50 cells were counted with the aid of a dissecting microscope.

**Figure 1** Cell survival curves of RIF-1 clones of different ploidy values following *in vitro* treatment with BCNU. Each point represents the survival value obtained from a single experiment. (■) clone 20 (tetraploid); (●) clone 16 (tetraploid); (□) clone 28 (diploid); (○) clone 23 (diploid).

**Figure 2** As for **Figure 1** except following *in vitro* treatment with MeCCNU.

### Results

### In vitro *sensitivity of RIF-1 clones to selected nitrosoureas*

The data showing the responses of RIF-1 clones to treatment with BCNU, MeCCNU and chlorozotocin are shown in Figures 1–3. For all three drugs, tetraploid clones 16 and 20 are consistently more resistant to treatment than diploid clones 23 and 28 (Similar data were also obtained for CCNU treatment confirming our previous findings).

Figure 3 As for Figure 1 except following *in vitro* treatment with chlorozotocin.

**Figure 4** Cell survival curves of RIF-1 clones following *in vitro* treatment with Nitrogen Mustard. Each point represents the survival value obtained from a single experiment (■) clone 20 (tetraploid); (●) clone 19 (tetraploid); (▲) clone tetraploid).

### In vitro *sensitivity of RIF-1 clones to the nitrogen mustards*

Figures 4, 5 and 6 show the responses of 3 tetraploid RIF-1 clones to treatment with nitrogen mustard, chlorambucil and aniline mustard. It can be seen that for all three drugs, clone 20 is markedly more resistant to *in vitro* treatment, over the dose range tested, than clones 16 and 19. (The same pattern of sensitivity was also observed when clones 16, 19 and 20 were treated with melphalan confirming our previous findings).

Although clone 20 is relatively resistant to treatment with nitrogen mustard itself, the shape of its dose-response survival curve is the same as that of clones 16 and 19; i.e. for all 3 clones there is an exponential decline in survival with increasing dose (Figure 4), as was obtained previously with melphalan. However, for chlorambucil and aniline mustard treatment, the survival curve for resistant clone 20 is not exponential like that of clones 16 and 19, but at high doses reaches a constant value (Figures 5, 6).

**Figure 5** As for **Figure 4** except following *in vitro* treatment with chlorambucil.

### Discussion

We have previously shown a wide variation in the responses of RIF-1 clonal subpopulations to both

**Figure 6** As for **Figure 4** except following *in vitro* treatment with Aniline Mustard.

*in vivo* and *in vitro* treatment with the alkylating agents melphalan and CCNU (Reeve *et al.*, 1983*b*). For melphalan, drug sensitivity was independent of the ploidy level of individual clones, but for CCNU treatment a clear relationship existed between ploidy and drug sensitivity with tetraploid and octoploid clones being markedly more resistant to drug treatment than diploid clones.

Several possible mechanisms of resistance to alkylating agents have been described including (a) reduced membrane permeability to the drug (e.g. Goldenberg *et al.*, 1970), (b) reduced drug transport (e.g. Elliott & Ling, 1981; Redwood & Colvin, 1980), (c) the the presence of enzyme(s) either to circumvent a specific metabolic block or to enhance the capacity for repair of alkylated DNA (e.g. Crathorn & Roberts, 1966; Roberts *et al.*, 1968), (d) increased cellular concentration of protective agents such as thiols that spare critical target sites from lethal injury by alkylation (e.g. Calcutt & Connors, 1963; Goldenberg, 1969; Hirono, 1960). In order to elucidate possible cellular mechanisms involved in the patterns of drug sensitivity described above we have exposed RIF-1 clones to selected nitrosoureas and nitrogen mustards which differ significantly in a variety of physicochemical and biological activities. Thus the nitrosoureas were selected on the basis of their different relative alkylating activity versus carbamoylating activity[1] and different lipophilicities[2] (Wheeler *et al.*, 1974; Wheeler, 1976). The nitrogen mustards were selected according to their different transport properties (Goldenberg *et al.*, 1970; Begleiter *et al.*, 1983; Begleiter & Goldenberg, 1983), lipophilicities and chemical reactivities (Workman *et al.*, 1976).

The results obtained in the present study show that RIF-1 clones of different ploidy level, when exposed *in vitro* to nitrosoureas, BCNU, MeCCNU and chlorozotocin, show the same pattern of sensitivity as that previously obtained for CCNU exposure (Reeve *et al.*, 1983*b*). This occurs despite the marked differences shown by these drugs in both relative alkylating/carbamoylating activity and lipophilicity (Wheeler *et al.*, 1974; Wheeler, 1976). Evidence has been presented that BCNU and CCNU are taken up into cells by passive diffusion (Begleiter *et al.*, 1977). This is also likely to be true for the similarly lipophilic MeCCNU, but may not necessarily apply to the more hydrophilic chlorozotocin, which contains a sugar moeity. Taken over all, it seems unlikely that differences in drug uptake would account for the ploidy dependent drug responses exhibited by RIF-1 clones to nitrosoureas.

As is the case with nitrosoureas, the RIF-1 clones show the same pattern of sensitivity to the different nitrogen mustards, i.e., aniline mustard, chlorambucil and nitrogen mustard itself, as was previously observed with melphalan (Reeve *et al.*, 1983*b*). This was true despite the differences in lipophilicity, chemical reactivity (Workman *et al.*, 1976) and perhaps most important, differences in cell transport properties of the nitrogen mustards.

If the comparative resistance of clone 20 to melphalan is a result of reduced efficiency of the amino acid transport system, as is seen with CHO and L1210 resistant variants (Redwood & Colvin, 1980; Begleiter *et al.*, 1983), one would not expect it also to be resistant to nitrogen mustard, which is actively transported by a different carrier mechanism, the choline transport system, (Goldenberg *et al.*, 1971) or to the nitrogen mustards known or predicted to enter the cell by passive diffusion, respectively chlorambucil (Begleiter & Goldenberg, 1983) and aniline mustard. Thus differences in drug transport can probably be ruled out.

[1]Nitrosoureas decompose at physiological conditions to yield chloroethyldiazonium hydroxide moieties, which can chloroethylate nucleophiles in nucleic acids, and isocyanates which can react with proteins only be carbamoylation.

[2]The lipophilicity of a drug measures its relative solubility in water and lipid and is important in determining the ease with which a drug crosses the cell membrane.

Although the pattern of sensitivity shown by RIF-1 clones in response to treatment with the nitrogen mustards is the same throughout, the shape of the dose-response curve for clone 20 clearly varies with the drug under study. For both melphalan and nitrogen mustard there is an exponential decline in survival with increasing dose; for both aniline mustard and chlorambucil the dose survival curves decrease to a constant value at high doses. The shape of the dose survival curve for clone 20 in response to treatment with aniline mustard and chlorambucil is similar to that obtained for chemotherapeutic agents which kill cells in one portion of the cell cycle, i.e., phase specific agents (Bruce *et al.*, 1966). One possible interpretation of our data is that the resistance of clone 20 to aniline mustard and chlorambucil reflects the sensitivity of this clone to these agents in only one part of the cell cycle.

On the basis of the results described here, it seems likely that a common mechanism may predominantly govern the responses of the RIF-1 clones to the various nitrosoureas, while a different common mechanism may determine their response to the various nitrogen mustards. Drug uptake is almost certainly not involved and thiol levels and DNA repair capacity appear to us to be the most likely possibilities. It is tempting to speculate, for example, that the ploidy dependence of nitrosourea may be the result of gene dosage for the repair enzyme $0^6$ methylguanine transferase, the suicide enzyme thought to repair the initial chloroethyl adduct which subsequently results in DNA cross-linking (Erikson *et al.*, 1980). We are currently examining the rate of DNA repair, together with levels of intercellular thiols.

A major problem in cancer therapy is the heterogeneous nature of human tumours and the role that this heterogeneity plays in the existence within a single neoplasm, of tumour cell populations with differing susceptibilities to therapeutic agents. Nitrosoureas, such as those used in the present study, are used in the treatment of a number of human tumours, with varying success. It is well established that human tumours can be heterogeneous with respect to chromosome number and DNA content. Our findings suggest that tumour cell ploidy may be an important factor in determining the susceptibility of human tumour cells to treatment with nitrosoureas.

### References

BEGLEITER, A. & GOLDENBERG, G.J. (1983). Uptake and decomposition of chlorambucil by LS178Y Lymphoblasts *in vitro*. *Biochem. Pharmacol.*, **32**, 535.

BEGLEITER, A., LAM, H.P. & GOLDENBERG, G.J. (1977). Mechanism of uptake of nitrosoureas by LS178Y lymphoblasts *in vitro*. *Cancer Res.*, **37**, 1022.

BEGLEITER, A., GROVER, J., FROESE, E. & GOLDENBERG, G.J. (1983). Membrane transport, sulfhydryl levels and DNA cross-linking in Chinese Hamster Ovary cell mutants sensitive and resistant to melphalan. *Biochem. Pharmacol.*, **32**, 293.

BRUCE, W.R., MEEKER, B.E. & VALERIOTE, F.A. (1966). Comparison of the sensitivity of normal haematopoietic and transplanted lymphoma colony-forming cells to chemotherapeutic agents administered *in vivo*. *J. Natl Cancer Inst.*, **37**, 233.

CALCUTT, G. & CONNORS, T.A. (1963). Tumour sulphydryl levels and sensitivity to nitrogen mustard merophan. *Biochem. Pharmacol.*, **12**, 819.

CRATHORN, A.R. & ROBERTS, J.J. (1966). Mechanism of the cytotoxic action of alkylating agents in mammalian cells and evidence for the removal of alkylating groups from deoxyribonucleic acid. *Nature*, **211**, 150.

ELLIOTT, E.M. & LING, V. (1981). Selection and characterization of Chinese hamster ovary cell mutants resistant to melphalan (L-phenylalanine mustard). *Cancer Res.*, **41**, 393.

ERIKSON, L., LAURANT, G., SHARKLEY, N.A. & KOHN, K.W. (1980). DNA cross-linking and mono-adduct repair in nitrosourea-treated human tumour cells. *Nature*, **288**, 727.

GOLDENBERG, G.J. (1969). Properties of LS178Y lymphoblasts highly resistant to nitrogen mustard. *Ann. N.Y. Acad. Sci.*, **163**, 936.

GOLDENBERG, G.J., VANSTONE, C.L., ISRAELS, L.G., ILSE, D. & BIHLER, I. (1970). Evidence for a transport carrier of nitrogen mustard in nitrogen mustard-sensitive and resistant LS178Y lymphoblasts. *Cancer Res.*, **30**, 2285.

GOLDENBERG, G.J., VANSTONE, C.L. & BIHLER, I. (1971). Transport of nitrogen mustard on the transport-carrier for choline in LS178Y lymphoblasts. *Science*, **171**, 1148.

HIRONO, I. (1960). Non-protein sulphydryl groups in the original strain and subline of the ascites tumour resistant to alkylating reagents. *Nature*, **186**, 1059.

REDWOOD, W.R. & COLVIN, M. (1980). Transport of melphalan by sensitive and resistant L1210 cells. *Cancer Res.*, **40**, 1144.

REEVE, J.G. & TWENTYMAN, P.R. (1983*a*). Clonal variation in the arrest, survival and growth of RIF-1 mouse sarcoma cells in the lungs of C3H mice. *Br. J. Cancer*, **47**, 833.

REEVE, J.G., WRIGHT, K.A. & TWENTYMAN, P.R. (1983*b*). Response to X-radiation and cytotoxic drugs of clonal subpopulations of different ploidy and metastatic potential isolated from RIF-1 mouse sarcoma. *Br. J. Cancer*, **47**, 841.

ROBERTS, J.J., CRATHORN, A.R. & BRENT, T.P. (1968). Repair of alkylated DNA in mammalian cells. *Nature*, **218**, 970.

TWENTYMAN, P.R., BROWN, J.M., GRAY, J.W., FRANKO, A.J., SCOLES, M.A. & KALLMAN, R.F. (1980). A new mouse tumour model system (RIF-1) for comparison of endpoint studies. *J. Natl Cancer Inst.*, **64,** 595.

WHEELER, G.P., BOWDEN, B.J., GRIMSLEY, J.A. & LLOYD, H.H. (1974). Interrelationships of some chemical, physicochemical, and biological activities of several 1-(2-Haloethyl)-1-nitrosoureas. *Cancer Res.*, **34,** 194.

WHEELER G.P. (1976). A review of studies on the mechanisms of action of nitrosoureas. *Am. Chem. Soc. Sym. Series,* **30,** 87.

WORKMAN, P., DOUBLE, J.A. & WILMAN, D.E.V. (1976). Enzyme activated anti-tumour agents-III. Hydrolysis of conjugates of p-hydroxyaniline mustard in aqueous solution. *Biochem. Pharmacol.,* **25,** 2347.